# Polymicrobial interactions influence *Mycobacterium abscessus* co-existence and biofilm forming capabilities

**DOI:** 10.3389/fmicb.2024.1484510

**Published:** 2024-11-25

**Authors:** Nishant Nandanwar, Geoffery Gu, Joy E. Gibson, Michael N. Neely

**Affiliations:** ^1^Division of Infectious Diseases, Department of Pediatrics, Children’s Hospital Los Angeles, Los Angeles, CA, United States; ^2^Department of Biological Sciences, University of Southern California, Los Angeles, CA, United States; ^3^Department of Pediatrics, Keck School of Medicine, University of Southern California, Los Angeles, CA, United States

**Keywords:** co-culture, polymicrobial interaction, coinfection, *Mycobacterium abscessus*, *Pseudomonas aeruginosa*, *Staphylococcus aureus*, biofilm formation

## Abstract

The lungs of patients with cystic fibrosis (CF) are vulnerable to persistent polymicrobial colonization by bacterial pathogens including *Pseudomonas aeruginosa, Staphylococcus aureus*, and the non-tuberculous mycobacterium (NTM) *Mycobacterium abscessus*. The polymicrobial milieu within the CF lung impacts individual species fitness, influences biofilm-forming capabilities, pathogenicity, production of virulence factors and even antimicrobial responses, all potentially compromising therapeutic success. Interaction studies among these CF pathogens are very limited, especially studies on the influences of *P. aeruginosa* and *S. aureus* on *M. abscessus* co-existence and virulence. Based on the little known thus far about coinfection of these pathogens, we hypothesize that the co-existence of *P. aeruginosa* and *S. aureus* alters *M. abscessus* virulence and phenotypic characteristics. We evaluated the direct (co-culture) and indirect (using supernatant) effects of *P. aeruginosa* and *S. aureus* on *M. abscessus* growth rate, biofilm formation, macrophage internalization and glycopeptidolipids (GPL) expression. Our observations indicate that *P. aeruginosa* and *S. aureus* exert a competitive behavior toward *M. abscessus* during direct contact or indirect interaction *in-vitro*, probably as is the case of polymicrobial infections in the lungs of patients with CF. This is the first report that demonstrates *S. aureus* inhibitory effects on *M. abscessus* growth and biofilm forming capabilities. Collectively, co-culture studies enhance our understanding of polymicrobial interactions during coinfection and can guide to establish better management of coinfections and treatment strategies for *M. abscessus*.

## 1 Introduction

Microbes in humans and animals mostly exist as polymicrobial communities of fungi, bacteria, and viruses sharing a common niche (Brogden et al., 2005). Microbial interactions in these common habitats may be uni- or bi-directional, supportive or competitive, and mediated by direct and/or indirect contact. Such interactions may alter virulence, antimicrobial resistance, and/or host immune responses (He and Shi, 2009; Peleg et al., 2010; Peters et al., 2012). Polymicrobial infections characterize the chronic and recurrent lung infections in patients with cystic fibrosis (CF), resulting in increased morbidity and death (Peters et al., 2012; Blanchard and Waters, 2019).

CF is an autosomal recessive genetic disorder, caused by inherited mutations in the cystic fibrosis transmembrane conductance regulator (CFTR) chloride ion channel (Davies et al., 2007). The lack of functional CFTR results in imbalances in chloride ion secretion outside the cell leading to cationic influx (McPherson et al., 1988). This dysfunction of mucociliary clearance causes accumulation of dehydrated and thick mucus that provides the ideal ground for bacterial growth (Bedrossian et al., 1976; Mortensen et al., 1993; Dodge and Turck, 2006). Due to these physiologic derangements, CF patients are less able to clear microbes. They develop polymicrobial infections with biofilms (Sibley et al., 2006) that favor microbial persistence and tolerance or resistance to antibiotics, making eradication difficult, if not impossible. The physical proximity between organisms of differing species and cross-species molecular signaling during polymicrobial infections can alter microbial phenotypic properties, virulence, pathogenicity and antimicrobial tolerance, thereby, exacerbating disease conditions in CF patients (Smith et al., 2015).

While many organisms such as *Streptococcus milleri* group (SMG) pathogens, *Burkholderia cepacia*, *Stenotrophomonas maltophilia, H. influenzae*, and *C. albicans* have been cultured from the bronchiolar lavage of CF patients (Peters et al., 2012), *Staphylococcus aureus* (*S. aureus*) and *Pseudomonas aeruginosa* (*P. aeruginosa*) are the most prominent species causing respiratory infections in patients with CF (Birmes et al., 2017). However, nontuberculous mycobacteria (NTM) are an emergent group, infecting up to 14% of patients with CF and chronic lung disease. *Mycobacterium abscessus* (*M. abscessus*) is the most common, rapidly growing, and highly antibiotic-resistant NTM that causes up to 20% of NTM disease (Prevots et al., 2010; Lee et al., 2015). Also according to the Cystic Fibrosis Foundation article, in the US in 2017, approximately 13% of people with CF had a positive culture for a NTM species and from 2010 to 2016, 20% of people with CF at least once grew NTM (Nontuberculous Mycobacteria (NTM) | Cystic Fibrosis Foundation, n.d.). Due to intrinsic and acquired antibiotic resistance, treatment of pulmonary *M. abscessus* consists of multi-drug therapy for one or more years, is effective only in about half of the patients, and is associated with numerous adverse effects (Chen et al., 2019; Kwak et al., 2019; Nandanwar et al., 2021, 2024).

Co-infection with *P. aeruginosa* and *M. abscessus* correlates with a more rapid decline in pulmonary function (Hauser et al., 2011; Qvist et al., 2015). A few co-culture studies demonstrate that *M. abscessus* can inactivate *P. aeruginosa* quorum sensing and outgrow *P. aeruginosa* (Birmes et al., 2017). Conversely, *P. aeruginosa* can alter *M. abscessus* biofilm formation (Rodríguez-Sevilla et al., 2018b, 2019). However, little else is known of the interactions between *M. abscessus* and *P. aeruginosa*, and to our knowledge there is no study that describes the interaction and impact of *S. aureus* (including both Methicillin-resistant *Staphylococcus aureus—*MRSA and Methicillin sensitive *Staphylococcus aureus—*MSSA) on *M. abscessus* virulence and pathogenesis despite documented coexistence in lung infections (Cullen et al., 2000; Hauser et al., 2011; Gilligan, 2014; Filkins and O’Toole, 2015).

We tested the hypothesis that interactions with *P. aeruginosa* and/or *S. aureus* might alter *M. abscessus* pathogenesis by affecting the growth pattern, virulence, and antimicrobial susceptibility. We report the effects of direct interactions arising from co-culture of live bacteria and indirect interactions using bacterial culture supernatant on phenotypic characteristics, pathogenicity, and virulence. This study enhances our understanding of the effect of polymicrobial environments on *M. abscessus* in patients with CF, with the long-term goal of improving *M. abscessus* therapy.

## 2 Materials and methods

### 2.1 Bacterial strains and culture conditions

All bacterial strains used in this study are listed in Supplementary Table S1.

We cultured *M. abscessus* ATCC 19977 (hereafter Mab) aerobically at 37°C in 7H9 Middlebrook (MB) (BD, Difco) broth supplemented with 0.2–0.5% glycerol, 0.05% Tween 80 and 10% ADC (albumin-dextrose-catalase) (BD, BD Biosciences) to an OD_600_ of 1.3–1.6. This culture was used to prepare stocks in MB media with 20% glycerol and stored at −80°C. These stocks were used as an initial inoculum to culture Mab in Muller Hinton (MH) media for all our co-culture experiments as MH media supported the growth of all the bacteria used in this study. Glycerol stocks of *P. aeruginosa* ATCC 27853 (hereafter referred to as PaATCC) and other strains of *P. aeruginosa* (PA collectively used for all the *P. aeruginosa* isolates used in this study, unless specified as PaATCC for ATCC 27853), and *S. aureus* (SA) (hereafter SA, used collectively for MRSA and MSSA) stored at −80°C were streaked on blood agar plates and a single colony was used to inoculate cultures in MH media for overnight incubation to be used as an inoculum for all the experiments. We used two different approaches for bacterial co-culture studies; (1) Direct co-culture—when Mab and other bacteria were cultured together in MH media, (2) Indirect method—Mab grown in presence of filtrate (or supernatant) of PA and SA prepared in MH media. For infection experiments we harvested logarithmic phase bacterial cells by centrifuging bacterial inoculum at 8,000 rpm for 5 min and resuspended the pellet in experimental media (DMEM+5% FBS) for infection in RAW264.7 murine macrophages. Any clump of Mab was removed by passing the bacterial suspension through a 28G syringe needle (BD Sciences) at least 15 times (Kim et al., 2019). After infection, we determined bacterial colony forming units (CFU) by plating 10-fold dilutions of the bacterial suspension in duplicate on selective MH or LB agar plates after 4–5 days of incubation at 37°C.

### 2.2 Selective agar plates for bacterial colony forming units quantification during direct co-culture

Colony counts were determined by the drop plating method on selective media agar plates (Rodríguez-Sevilla et al., 2018b). Mab colonies were selected on Columbia Colistin Nalidixic Acid (CNA) agar plates when co-cultured with PA (Rodríguez-Sevilla et al., 2018b) and on vancomycin (2 mg/mL) when co-cultured and with SA. PA colonies were selected on MacConkey agar plate (Rodríguez-Sevilla et al., 2018b) that inhibits Mab and SA growth. SA when co-cultured with PaATCC were selected on CNA agar plates and on normal MH plates during co-culture with Mab as SA colonies appeared following day of plating while Mab colonies took 4–5 days to grow.

### 2.3 Supernatant preparation

Bacterial supernatants after culturing PA isolates and SA in MH media were prepared with slight modifications from Beaudoin et al. (2017). A single colony of each PA strain and SA was inoculated separately in 5 mL MH media in 50 mL tubes and incubated overnight at 37°C in a shaking incubator. A 100 μL aliquot of this overnight culture was then added to a fresh 100 mL of MH media and incubated at 37°C in a shaking incubator until reaching an OD600_nm_ of 2.0–2.2. Following growth, bacterial cells were pelleted by centrifugation at 8,000 rpm for 15 min. We collected the supernatant and sterilized it by passing it through a 0.22 μm filter. Total protein concentration of the supernatant was determined using bicinchoninic acid (BCA) kit and the concentrations were maintained for all the experiments. We checked the sterility of supernatants by plating 1 mL of supernatant on agar plates (200 μL/plate). For all the indirect contact experiments, we diluted each of the supernatants from different strains to 1:1 with MH media. To account the use of supernatant (spent media) for our indirect contact experiments we used MH media diluted 1:1 with 1xPBS as a control. After filtration, supernatant was autoclaved in an autoclave for preparation of heat inactivated supernatant.

### 2.4 Growth pattern of Mab during co-culture

We determined the growth pattern of Mab in co-culture with other CF pathogens such as PA and SA in MH broth media with modifications from Birmes et al. (2017) and Rodríguez-Sevilla et al. (2018b). For the direct co-culture method, we diluted overnight cultures of Mab to 5 × 10^5^ or 5 × 10^7^ CFU/ml separately and PA and SA to 10^3^ CFU/mL respectively, in 10 mL of MH media and incubated them at 37°C and 200 rpm in a shaker incubator for 120 h. The different inoculum sizes controlled for differences in growth rates. The monocultures of Mab, PA and SA served as controls. Every 24 h, aliquots were serially diluted and plated to enumerate viable CFU. For the indirect co-culture method, we grew Mab (5 × 10^5^ or 5 × 10^7^ CFU/ml) in supernatants of PA and SA diluted with MH media (1:1). As a control, Mab was also grown in MH media diluted with 1xPBS (1:1). All growth curve experiments were continued for 120 h.

### 2.5 Biofilm formation capability of Mab

We assessed the biofilm forming capability of Mab in the presence and/or absence of PA and SA as described by Rodríguez-Sevilla et al. (2018a, 2018b) with modifications. For direct contact, overnight cultures of PA and SA grown in MH media were diluted to a final concentration of 10^3^ CFU/mL. Mab overnight cultures were diluted to the final concentration of 5 × 10^5^ CFU/ml in the bacterial suspension. 200 μL of this bacterial suspension (mixture of Mab and other bacteria) was added in triplicate to a 96 well plate and covered with a permeable membrane and incubated at 37°C for 72 h without shaking. Single species inoculated in separate wells served as controls and wells with only MH media served as sterility controls.

For indirect contact, the diluted supernatants of PA and SA prepared in MH media were used to adjust Mab to a concentration of 5 × 10^5^ CFU/ml in 96 well plates same as above. In each experiment the initial count of Mab was determined by diluting and plating the initial cultures before incubation.

### 2.6 Quantification of biofilm forming capability

#### 2.6.1 CFU counts to quantify bacterial biomass in biofilm

Bacterial biofilm biomass in co-culture (both direct and indirect contact) after 72 h was quantified with CFU counts as described by Rodríguez-Sevilla et al. (2018a, 2018b) with modifications. After 72 h of incubation, the supernatant from all the wells was removed and the biofilm settled at the bottom was resuspended in 250 μL of saline, serially diluted and plated on selective agar plates to count CFUs of biofilm biomass. We counted Mab colonies after 4–5 days on CNA agar when co-cultured with PA and on Vancomycin agar plates when co-cultured with SA. PA colonies were counted on MacConkey agar plates.

#### 2.6.2 Biofilm quantification using crystal violet staining

We also quantified the biofilm forming capability of Mab during indirect contact with other pathogens using CV staining in 96-well flat-bottomed plates as described previously with some modifications (Nandanwar et al., 2014; Sousa et al., 2015; Belardinelli et al., 2021; Chakraborty et al., 2021). Briefly, after incubation of plates at 37°C for 72 h, we removed the spent media from each well and washed the wells once with distilled water to remove non-adherent bacteria, and air-dried the plate. The biofilm was fixed with 99% methanol for 15 min, which we then aspirated and air-dried a second time. We stained the bacterial biofilm with 0.1% CV solution for 30 min and then washed the plate three times with distilled water and air-dried a third time. The CV stain was re-solubilized in ethanol: acetone (80:20) solution and absorption was measured at OD570 nm in the ELISA reader.

### 2.7 Bacterial internalization of RAW264.7 macrophages

We obtained RAW264.7 murine macrophages from the American Type Culture Collection (ATCC) and cultivated them using in Dulbecco’s modified Eagle media (DMEM, Corning) media supplemented with 10% Fetal bovine serum (FBS), 1% penicillin/streptomycin in a humidified CO_2_ incubator at 37°C, as recommended. Infection experiments to determine if the direct contact of bacterial organisms affect the internalization of Mab in macrophages were performed using infection assay with some modifications (Roux et al., 2016; Kim et al., 2017, 2019; Nandanwar et al., 2021). RAW264.7 cells were seeded in 24-well plates and were coinfected with Mab in presence/absence of PaATCC and SA respectively, at a multiplicity of infection (MOI) of 10 (for Mab) and 5 (for PaATCC and SA) for 2 h at 37°C with 5% CO_2_ (Tian et al., 2019; Kim et al., 2022). Later, we washed the infected RAW264.7 cells with DMEM three times to eliminate extracellular bacteria. Fresh experimental media containing Amikacin 200 μg/mL, which is bactericidal for all tested organisms, was added, and the cells were incubated for an additional 2 h to kill residual attached and planktonic extracellular bacteria. After 2 h of antibiotic treatment we removed the medium containing Amikacin and washed the cells again three times with DMEM. RAW264.7 cells were lysed with 0.5% Triton-X-100 prepared in saline. The lysates were diluted in saline and plated onto relevant agar plates to count CFUs of internalized bacteria. To check internalization of Mab under indirect contact we cultured Mab in presence of supernatant from PaATCC and SA (diluted 1:1 with MH media) separately and performed the infection experiment as described above. The viability of infected macrophages compared to uninfected cells was evaluated by Trypan blue and was comparable before and after infection.

### 2.8 Glycopeptidolipids extraction and thin layer chromatography

Lipid extraction from Mab after indirect co-culture was extracted using the chloroform-methanol method (Folch et al., 1957; Billman-Jacobe et al., 1999; Tatham et al., 2012) with some modifications, followed by thin-layer chromatography (TLC). Briefly, we cultured Mab in supernatant (diluted 1:1 with MH media) of PA and SA separately at 37°C in a shaking incubator for 120 h and used these cultures for GPL isolation. After incubation, the bacterial suspension was centrifuged at 4,700 × g for 10 min, the pellet was washed with 1xPBS and centrifuged again at the same speed. Equal amounts of the pellets (wet weight) for each sample were then dissolved in 2:1 chloroform/methanol (20 μL/mg of wet weight) and kept on a rocking shaker overnight at room temperature. The following day, we briefly sonicated the suspension four times each for 15 s and then centrifuged at 18,400 × g for 10 min to remove insoluble material. The chloroform/methanol supernatant was placed in a fresh tube and 0.2 volume of 0.9% NaCl was added and mixed vigorously by vortexing. Later, we centrifuged the mixture at 1,150 × g for 5 min. The organic phase containing GPL was collected and dried to evaporate any traces of alcohol. The dried lipid pellet was redissolved in chloroform/methanol (2:1), and 15 μL (300 μg) of each sample was loaded onto aluminum-backed silica gel TLC plate and irrigated with chloroform/methanol (100:7). The TLC plate was removed, dried, and sprayed with 0.1% resorcinol in 40% sulfuric acid, and glycolipids were detected by charring the plate at 140°C.

### 2.9 Pyocyanin extraction from *Pseudomonas aeruginosa*

Since PA isolates are known to produce a secondary metabolite pyocyanin with antimicrobial properties (Gonçalves and Vasconcelos, 2021), we checked pyocyanin production in the isolates used in this study by extracting pyocyanin as described previously (Essar et al., 1990; Schmidt et al., 2023). Briefly, we cultured PA isolates in 5 mL MH media for at least 24 h and then centrifuged the bacterial suspension to pellet the bacteria. To the clear supernatant we added chloroform at 50% vol/vol. Samples were then vortexed vigorously and centrifuged at 6,000 × g for 5 min to separate the organic phase (lower layer containing chloroform) that was removed and transferred to a fresh tube and then 20% the volume of 0.1 N HCl was added and mixed vigorously. We then centrifuged the samples again at 8,000 rpm for 5 min. After centrifugation, the aqueous fraction (upper layer) was aliquoted in a 96-well plate and absorbance was measured at 520 nm. Pyocyanin concentration as μg/ml, was obtained by multiplying the OD520 nm by 17.072, as described previously (Essar et al., 1990; Schmidt et al., 2023).

### 2.10 Growth pattern and biofilm formation capabilities of Mab in presence of *Pseudomonas aeruginosa* pyocyanin

*P. aeruginosa* produces different exoproducts that demonstrate antimicrobial and antibiofilm properties against other pathogens during co-infection, one such exoproduct is pyocyanin (Ozdal, 2019; Gonçalves and Vasconcelos, 2021). Hence, we checked the growth pattern and biofilm forming capabilities of Mab in presence of pyocyanin (pure pyocyanin procured from Sigma-Aldrich). We cultured Mab in MH media supplemented with different concentrations of pyocyanin and compared it with the growth of Mab in MH media supplemented with equivalent volume of Dimethyl sulfoxide (DMSO) as pyocyanin was dissolved in DMSO, serving as a control. We also determined biofilm formation by Mab in presence of pyocyanin followed by CV staining as described in above sections.

### 2.11 Statistical analysis

We performed statistical analyses using Prism Software (GraphPad version 9.0.0), and a *p*-value less than or equal to 0.05 was considered statistically significant. Normal distribution of the data was assessed using the Shapiro–Wilk test. Statistical significance was assessed by one way ANOVA-test for comparison of more than two conditions in an experiment. Independent Student’s *t*-test was used for comparison between two conditions.

## 3 Results

### 3.1 PaATCC and SA inhibit Mab growth in co-culture

**Figure 1:**
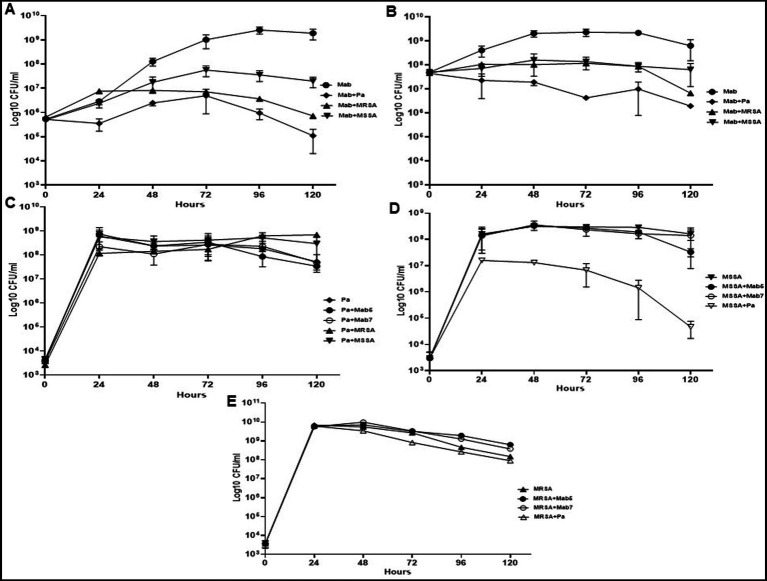
Growth pattern of Mab in co-culture (direct contact) with PaATCC and SA. **(A)** The presence of PaATCC and SA (collectively used for MRSA and MSSA) in co-culture with Mab (5 × 10^5^ CFU/ml) inhibited Mab growth when compared to the Mab monoculture. PaATCC in co-culture with Mab demonstrated the maximum inhibitory effect on Mab growth. **(B)** Even with the higher starting inoculum of Mab (5 × 10^7^ CFU/ml), the maximum growth inhibition of Mab was observed during co-culture with PaATCC. **(C)** On the contrary, the growth pattern of PaATCC was unaffected by the presence of Mab (higher and lower inoculum) and SA. **(D,E)** PaATCC also inhibited the growth of MSSA and MRSA in co-culture compared to the monoculture. However, both MRSA and MSSA were unaffected by the presence of Mab in co-culture. The starting inoculum for PaATCC, MRSA, and MSSA was 10^3^ for each, respectively (Mab5, represents 5 × 10^5^ CFU/ml and Mab7, represents 7 × 10^7^ CFU/ml respectively). Growth curve experiments were performed in MH media as it supported the growth of all the participating bacteria in a co-culture. The graph represents the mean data and standard deviation of three independent experiments plated each time in duplicate. Pa represents PaATCC (please check Supplementary Tables S2A–C for statistical analysis of growth pattern of Mab, PaATCC, MSSA, and MRSA at different time points).

Both PaATCC and SA significantly inhibited Mab growth in co-culture (direct contact) compared to the Mab monoculture (Figure 1A). PaATCC most strongly inhibited Mab growth, and MRSA was more inhibitory compared to MSSA in co-culture (Figure 1A). To account for different growth rates among these organisms we also tested higher inoculum of Mab during co-culture. Even at the higher Mab starting inoculum (5 × 10^7^ CFU/ml), PaATCC and SA significantly inhibited Mab growth compared to Mab monoculture (Figure 1B).

**Figure 2:**
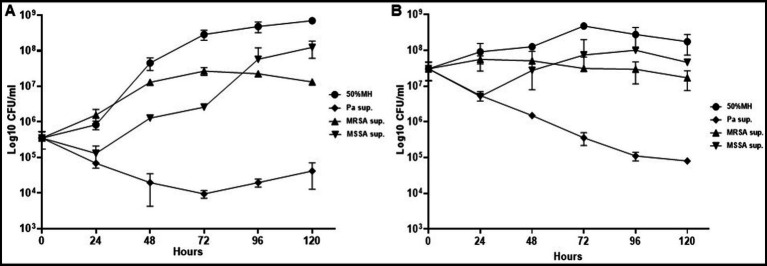
Growth pattern of Mab in supernatant (indirect contact) of PaATCC and SA. **(A,B)** Indirect contact of PaATCC, MRSA, and MSSA with Mab using supernatant prepared in MH media inhibited the growth of Mab (even at lower 5 × 10^5^ and higher 5 × 10^7^ CFU/ml of Mab starting inoculum). PaATCC supernatant demonstrated the maximum growth inhibitory effect on Mab growth. Supernatant prepared in MH media were diluted to 1:1 with MH media for the experiment and MH media diluted 1:1 with 1XPBS was used as a control for Mab growth (Pa sup. Represents PaATCC supernatant). The graph represents the mean data and standard deviation of three independent experiments plated each time in duplicate. Sup., supernatant (please check Supplementary Table S3 for statistical analysis of growth pattern of Mab under different conditions at different time points).

Conversely, PaATCC growth was not affected significantly by either Mab or SA (Figure 1C). PaATCC also inhibited the growth of SA, although more strongly and significantly for MSSA than MRSA (Figures 1D,E). Lastly, Mab did not significantly affect the growth of and SA (Figures 1D,E). Collectively, the presence of PaATCC and SA inhibited Mab growth, with a predominant inhibition observed in the presence of PaATCC. Also, during indirect co-culture, PaATCC supernatant significantly inhibited Mab growth more predominantly than SA supernatant compared to the control (Figures 2A,B). In summary, PaATCC and SA inhibited Mab directly and indirectly. But SA inhibited Mab to a lesser extent than PaATCC in direct co-culture and indirect interaction. Neither SA nor Mab affected PaATCC growth significantly during direct co-culture.

**Figure 3:**
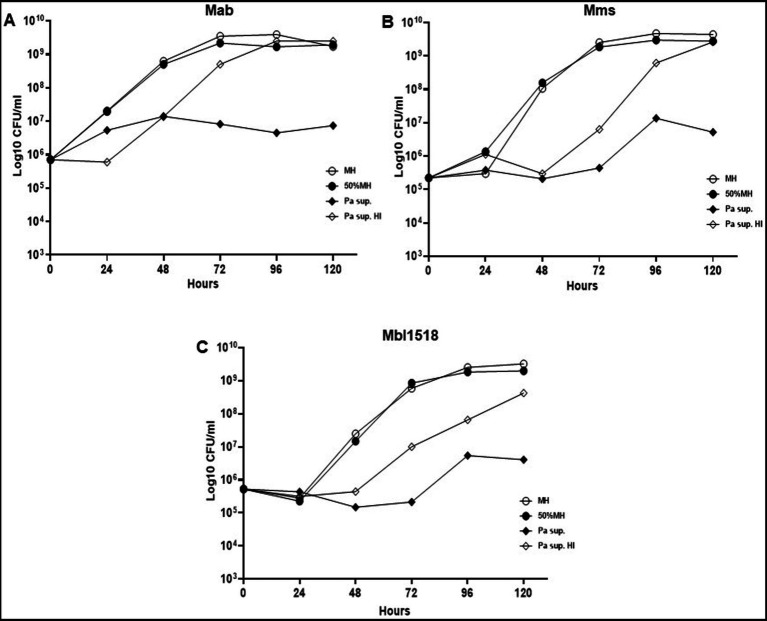
Heat inactivated supernatant does not inhibit *M. abscessus* growth. **(A)** The indirect contact of PaATCC inhibited Mab growth compared to the MH media control. However, the heat inactivated supernatant did not show growth inhibitory effect on Mab. **(B,C)** PaATCC supernatant also inhibited the growth of *M. abscessus* strains of other sub species such as the *Mab. massiliense* (Mms) and *Mab. bolletii*. 1518 (Mbl1518) compared to MH media control. However, the heat inactivated supernatant did not affect the growth of Mms and Mbl1518 (Pa sup. Represents PaATCC supernatant). The graph represents the mean data and standard deviation of two independent experiments plated each time in duplicate. Pa, PaATCC; sup., supernatant; HI, heat inactivated supernatant (please check Supplementary Table S4 for statistical analysis for **A–C**).

Since PaATCC significantly affected Mab growth, even during indirect contact, we also checked if the same is observed for strains of other sub. species of Mab. PaATCC also inhibited the growth of Mms and Mbl1518 strains, indicating that PaATCC has an inhibitory effect on growth of other *M. abscessus* sub. species (Figures 3A–C). The heat-inactivation of supernatant of PaATCC, however, did not inhibit the growth of Mab strains as strongly and significantly as the normal supernatant (supernatant without heat inactivation), although it did affect the growth of Mab strains to certain extent (Figures 3A–C). Hence, certain heat labile metabolites are produced by PaATCC that inhibit Mab strains growth compared to MH media control (Figures 3A–C) and heat inactivation reduces the inhibitory effect of PaATCC supernatant by affecting heat liable metabolites present in the supernatant.

### 3.2 PaATCC and SA inhibit *in-vitro* biofilm forming capabilities of Mab in a co-culture

**Figure 4:**
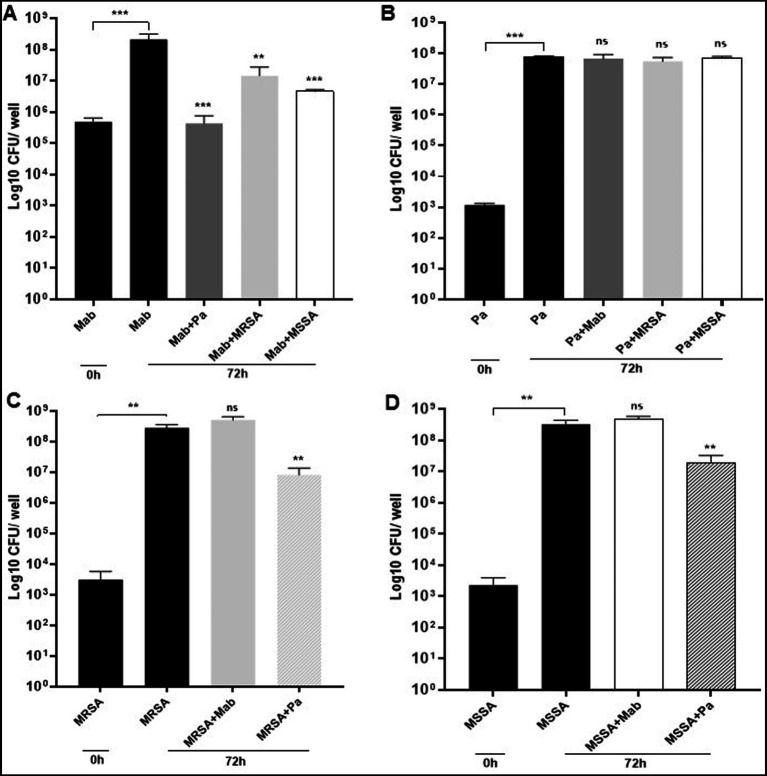
Mab biofilm forming capabilities in co-culture. **(A)** Mab biofilm forming capabilities were significantly altered or reduced in the presence of PaATCC, MRSA, and MSSA in co-culture after 72 h of incubation compared to the Mab monoculture. **(B)** The presence of Mab, MRSA, and MSSA in co-culture did not affect PaATCC biofilm forming capabilities after 72 h of co-culture. **(C,D)** Mab presence did not affect MRSA and MSSA biofilm. However, PaATCC in co-culture with MRSA and MSSA significantly reduced their biofilm forming capabilities after 72 h of incubation compared to the monoculture. The black solid bars represent controls that demonstrated significant biofilm formation by monoculture after 72 h of incubation compared to 0 h. The data represents mean with standard deviation from three independent experiments performed in triplicate each time. Statistics were performed using one-way ANOVA for multiple comparisons. Statistical significance is represented in figures by asterisks. ***p < 0.0005 and **p < 0.005. ns, non-significance; Pa, PaATCC.

We found that the presence of PaATCC and SA in co-culture significantly inhibited biofilm forming capabilities of Mab as there was significant reduction in the Mab biofilm biomass after 72 h of incubation in co-culture compared to the Mab monoculture biofilm biomass (Figure 4A). On the other hand, PaATCC biofilm forming capabilities did not change during co-culture with Mab or SA when compared to PaATCC monoculture biofilm after 72 h (Figure 4B). Apart from Mab, PaATCC also significantly inhibited MRSA and MSSA biofilm in co-culture compared to monoculture, while being unaffected by the presence of Mab in co-culture after 72 h of incubation (Figures 4C,D).

**Figure 5:**
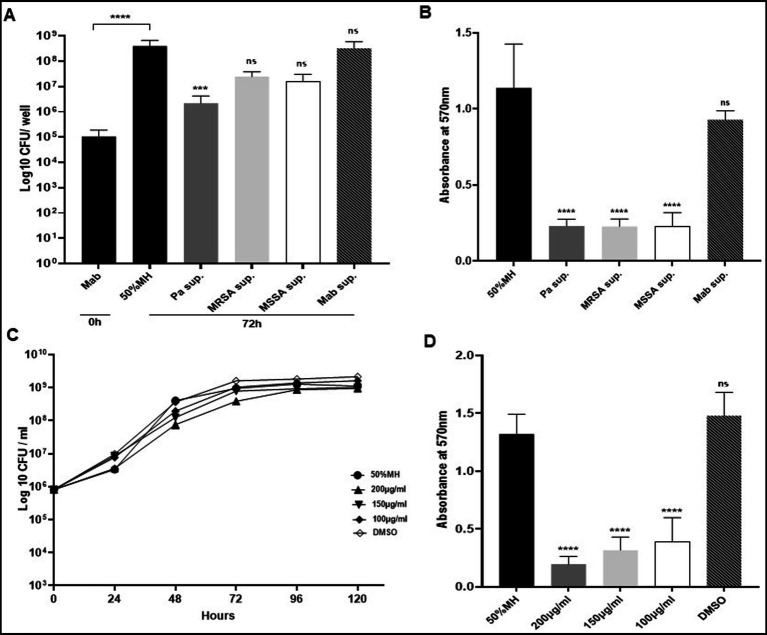
Biofilm capabilities of Mab during indirect contact (using supernatant). **(A)** Mab biofilm forming capability was significantly reduced in the presence of PaATCC supernatant. Mab biofilm also reduced in presence of MRSA and MSSA supernatant but was not significant compared to the controls (Mab biofilm in diluted MH media and Mab supernatant respectively). Biofilm biomass by CFU counts was determined after 72 h of incubation. The black solid and black streaked bars represent controls that demonstrated significant biofilm formation after 72 h compared to 0 h (Pa sup. Represents PaATCC supernatant). **(B)** Biofilm forming capabilities as determined by CV staining of biofilm after 72 h revealed that Mab biofilm reduced significantly in presence of PaATCC, MRSA, and MSSA supernatant compared to the Mab biofilm in MH media and Mab supernatant used as controls. **(C)** Mab when grown in media supplemented with pyocyanin did not show any growth inhibition compared to the control (Mab grown in diluted MH media). (Equal volume of DMSO was used as a control as Pyocyanin was dissolved in DMSO) (please check Supplementary Table S5 for statistical analysis for **C**). **(D)** Pyocyanin significantly inhibited biofilm formation by Mab when compared to the media and DMSO controls. The growth curve data represents the mean and standard deviation of two independent experiments plated each time in duplicate. Biofilm forming assay was performed three times, each with at least six replicates each time. One-way ANOVA for multiple comparisons. Statistical significance is represented in figures by asterisks. ****p < 0.0001, ***p < 0.0005, ns, non-significance; Sup., supernatant.

As observed for the direct contact, the biofilm biomass of Mab was significantly reduced after 72 h of incubation in presence of PaATCC supernatant compared to the MH media control (Figure 5A) indicating that even the indirect interactions of PaATCC can inhibit Mab biofilm. Mab biofilm biomass was also reduced in SA supernatant but was not significant compared to the MH media control (Figure 5A). However, CV staining after 72 h of incubation demonstrated significant inhibition of Mab biofilm in supernatant of PaATCC and SA compared to the MH media control (Figure 5B).

Besides using diluted MH media (diluted 1:1 with 1xPBS) as our control, we also determined biofilm forming capabilities of Mab in supernatant prepared by culturing Mab in MH media as a control to match the conditions of using spent media (supernatant) for our indirect contact approach. We observed comparable biofilm formation by Mab in MH media control and Mab supernatant (Figures 5A,B; Supplementary Figure S1). This indicates that the reduction of Mab biofilm is due to the antibiofilm properties of extracellular metabolites produced by PaATCC and SA that inhibit biofilm forming capabilities of Mab and not due to the depleted nutrients in the supernatant.

In summary, PaATCC and SA directly and indirectly reduce the biofilm formation capabilities of Mab.

### 3.3 Pyocyanin produced by *Pseudomonas aeruginosa* inhibits Mab biofilm

We observed that growth pattern of Mab was not affected significantly when grown in MH media supplemented with pyocyanin (Figure 5C). However, the biofilm formation capabilities of Mab were significantly reduced in the presence of pyocyanin compared to the media control (Figure 5D). We used pyocyanin because it is one of the few commercially available metabolites produced by PA isolates with known antimicrobial properties and even inhibits biofilm formation by other pathogenic microbes during coinfection (Hauser et al., 2011; Korgaonkar et al., 2013). We observed differential expression of pyocyanin by PA isolates used in this study (Supplementary Figure S2A). We also found that PaATCC produced a higher amount of pyocyanin when co-cultured with Mab compared to PaATCC monoculture (Supplementary Figure S2B).

### 3.4 PaATCC enhances Mab internalization in RAW264.7 macrophages during coinfection

**Figure 6:**
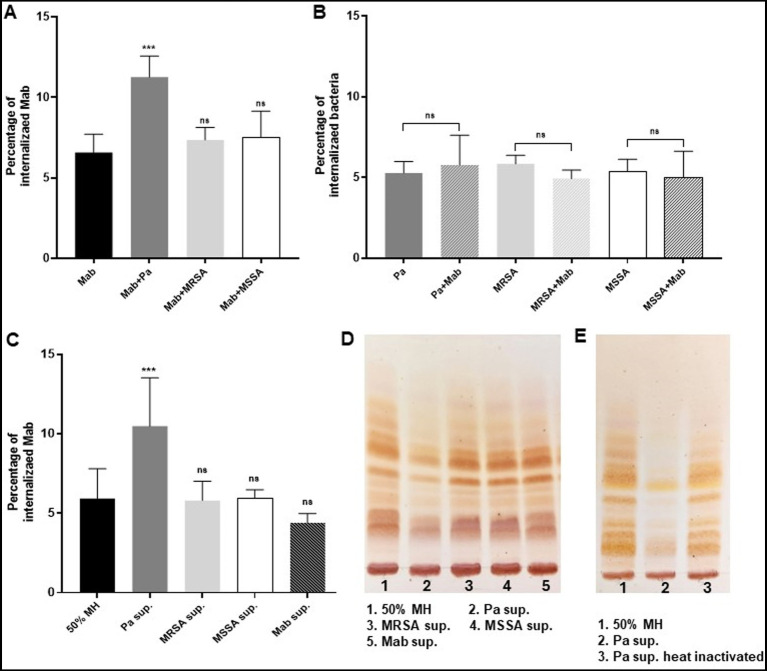
Presence of PaATCC during coinfection enhances internalization of Mab in RAW264.7 macrophages. **(A)** Mab demonstrated enhanced and significant internalization in RAW264.7 macrophages when coinfected (direct contact) with PaATCC compared to the Mab alone infection. However, the coinfection of Mab with MRSA and MSSA did not affect the internalization of Mab in RAW macrophages. **(B)** Internalization of PaATCC, MRSA and MSSA during coinfection with Mab did not show significant difference compared to monoculture infection. **(C)** Mab grown in Pa supernatant (indirect contact) also demonstrated significant internalization in RAW264.7 macrophages compared to the controls (Mab grown in diluted MH media and Mab supernatant respectively). However, Mab cultured in MRSA and MSSA supernatant did not show any affect on Mab internalization in macrophages compared to the controls (Pa sup. Represents PaATCC supernatant). **(D)** Lipid isolation and TLC demonstrated lower expression of GPL in Mab grown in PaATCC supernatant compared to the controls (Mab grown in diluted MH media and Mab supernatant). MRSA and MSSA supernatant did not affect GPL expression in Mab. **(E)** Mab grown in heat inactivated PaATCC supernatant showed GPL expression compared to the normal PaATCC supernatant. All the infection data represents the mean and standard deviation of three independent experiments performed in triplicate each time. The TLC is the representative image of three different experiments. One-way ANOVA for multiple comparisons for infection assay and Student’s *t* test for comparing two groups was used. Statistical significance is represented in figures by asterisks. ***p < 0.0005. ns, non-significance.

We observed that the presence of PaATCC during co-infection resulted in higher internalization of Mab in RAW264.7 macrophages compared to Mab infection alone (Figure 6A). However, no significant difference was observed during co-infection with SA compared to Mab-only infection in macrophages (Figure 6A). Mab on contrary, did not affect the internalization capabilities of PaATCC and SA during co-infection compared to experiments without Mab (Figure 6B). As observed for the direct contact during co-infection, Mab grown in the presence of PaATCC supernatant (indirect contact) also showed higher internalization in RAW264.7 macrophages compared to the controls (Mab grown in diluted MH media and in Mab supernatant). In contrast, Mab grown in the presence of SA supernatant did not affect the internalization of Mab in macrophages (Figure 6C).

In summary the PaATCC directly and indirectly enhanced internalization of Mab in RAW264.7 macrophages, while SA did not affect the internalization of Mab in RAW264.7 macrophages either directly or indirectly.

### 3.5 PaATCC alters GPL expression in Mab

Since the presence of PaATCC significantly enhanced the internalization capabilities of Mab in RAW264.7 macrophages, we also performed GPL isolation to check if PaATCC alters GPL expression in Mab during indirect contact using supernatant, as the lower expression of GPL by Mab is associated with higher internalization of Mab in macrophages (Nandanwar et al., 2021). Indeed, thin layer chromatography (TLC) analysis demonstrated lower GPL expression in Mab grown in PaATCC supernatant compared to the controls (Mab grown in MH media and Mab supernatant) as shown in Figure 6D. However, GPL expression in Mab did not alter when grown in SA supernatant (Figure 6D). Since PaATCC supernatant altered GPL expression, we also checked Mab GPL expression after exposure to heat-inactivated PaATCC supernatant and observed that the GPL production was normalized in Mab grown in heat-inactivated PaATCC supernatant (Figure 6E). Therefore, we can hypothesize that heat labile extracellular compounds are produced by PaATCC strain that alter GPL expression in Mab. We were unable to isolate GPL from Mab grown in co-culture with PaATCC and SA; hence GPL determination was possible only in supernatant experiments.

### 3.6 Different *Pseudomonas aeruginosa* isolates inhibit Mab growth and biofilm

**Figure 7:**
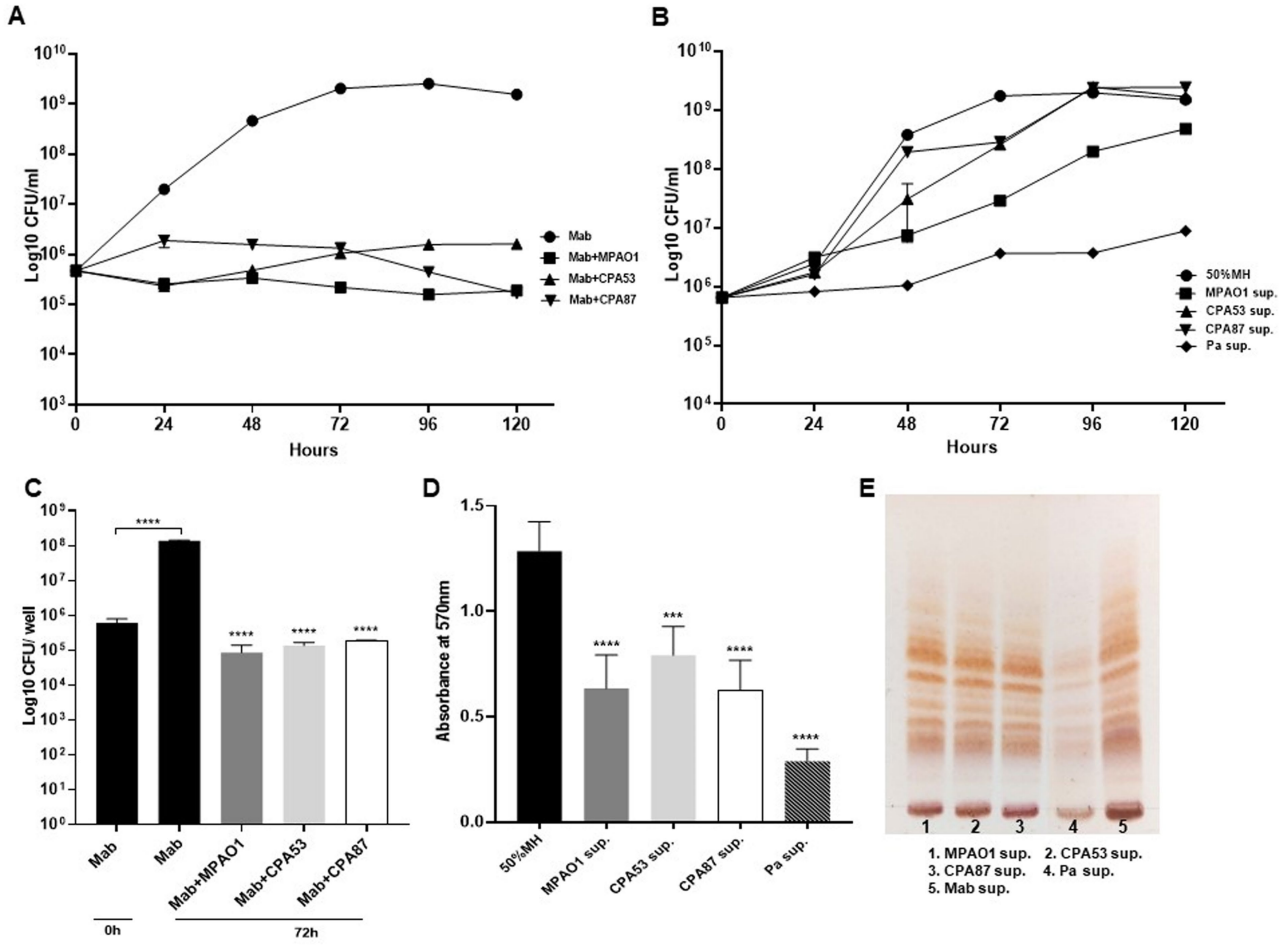
Mab growth pattern, biofilm forming capabilities and GPL expression during direct and indirect contact with other PA. **(A,B)** Mab growth pattern of Mab during direct contact and in presence of supernatant from other PA isolates. Mab growth was significantly inhibited during direct contact but did not change much with supernatant of other PA isolates other than MPAO1 as compared to the Mab growth in PaATCC supernatant (shown here as a control) (Pa sup. Represents PaATCC supernatant) (please check Supplementary Tables S6, S7 for statistical analysis of **A,B** respectively). **(C,D)** Significant inhibition in the biofilm forming capacity of Mab was observed during direct and indirect contact of Mab with other PA isolates compared to Mab biofilm formed after 72 h in diluted MH media used as a control. **(E)** Growth of Mab in supernatants of other PA did not affect the GPL expression in Mab as compared to the control (Mab grown in Mab supernatant was taken as a control). The growth curve data represents the mean and standard deviation of two independent experiments plated each time in duplicate. Biofilm forming assay was performed three times, each with at least six replicates each time. TLC is the combined representative image of three different experiments. Statistics were performed using one-way ANOVA for multiple comparisons for biofilm assay. ****p < 0.0001, ***p < 0.0005. Sup., supernatant.

Since we found Mab growth, biofilm formation, and GPL expression to be significantly altered by PaATCC during direct and indirect interaction, we also assessed if other *P. aeruginosa* isolates (*P. aeruginosa* reference strain MPAO1 and two *P. aeruginosa* clinical isolates, CPA53 and CPA87 respectively) also affected Mab characteristics. The direct co-culture of Mab with other isolates of *P. aeruginosa* also significantly inhibited Mab growth (Figure 7A). However, during indirect contact, Mab growth was inhibited more in the supernatant of MPAO1 compared to CPA53 and CPA87. Although it did not affect the growth as strongly as observed for PaATCC (used as a control here) as shown in Figure 7B. During both direct and indirect contact of Mab with MPAO1, CPA53, and CPA87, Mab biofilm forming capability was significantly inhibited (Figures 7C,D) as determined by CFU counts of biofilm biomass and CV staining. Interestingly, Mab GPL expression did not change during growth in supernatants of these isolates compared to the control (Mab supernatant) as shown in Figure 7E.

### 3.7 Mab revives its phenotypic characteristics when grown as monoculture after pre-exposure to *Pseudomonas aeruginosa* isolates

**Figure 8:**
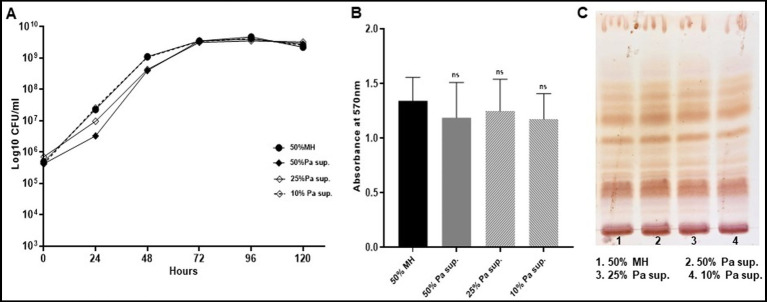
Mab revives its normal growth pattern, biofilm forming capabilities and GPL expression. Mab was grown in different concentrations (50, 25, and 10%) of PaATCC supernatant diluted with MH media and then centrifuged to pellet the bacteria. This pellet was resuspended in 1xPBS and used as an inoculum for growth curve, biofilm assay and for cultures to isolate GPL. Mab grown in diluted MH media (1:1 with 1xPBS) was used as a control. **(A)** Mab pretreated with PaATCC supernatant at different concentrations when grown in fresh media without any PaATCC supernatant revived its growth pattern comparable to the controlled growth condition (growth in diluted MH media) (please check Supplementary Table S8 for statistical analysis of **A**). **(B)** No significant difference was observed in the biofilm forming capabilities of Mab pretreated with PaATCC supernatant compared to the control (Mab grown in diluted MH media) after 120 h of incubation. **(C)** Lipid extraction and TLC of Mab grown in normal media after pretreatment with PaATCC supernatant did not show any significant difference in the expression of GPL as compared to the control (Mab grown in diluted MH media). The growth curve data represents the mean and standard deviation of three independent experiments plated each time in duplicate. The TLC is the representative image of three different experiments. Statistics were performed using one-way ANOVA for multiple comparisons. ns, non-significance; Sup., supernatant.

Since the direct and indirect contact of PaATCC with Mab significantly affected Mab growth pattern, biofilm formation we also evaluated if pre-exposure of Mab to PaATCC (using supernatant) durably changes Mab phenotypic characteristics. We grew Mab in presence of PaATCC supernatant, diluted with MH media to different concentrations (50, 25, and 10% respectively). After incubation, we centrifuged and washed Mab with 1xPBS before setting up experiments to check the growth pattern, biofilm formation and GPL expression. We observed that Mab pre-exposed to PaATCC supernatant and then grown in normal MH media, regains its normal growth pattern (Figure 8A) with no significant difference compared to the media control. Moreover, Mab also retains its biofilm formation capabilities and GPL expression once it is not under the stress of PaATCC supernatant (Figures 8B,C). These observations indicate that PaATCC alters Mab phenotypic characteristics in a reversible manner.

Similar observations were also observed after pre-exposure of Mab to supernatant of MPAO1, CPA53, and CPA87, wherein, Mab regained its normal growth pattern, biofilm formation and GPL expression once the stress of supernatant is replaced by normal MH media (Supplementary Figures S3A–C).

## 4 Discussion

The lungs of patients with CF are colonized with polymicrobial communities. The dominant organisms are PA and SA, and both are associated with morbidity and mortality (Vandeplassche et al., 2019). With treatment often focused on these pathogens, other species emerge such as Mab, a rapidly growing NTM found in many patients with CF (Vandeplassche et al., 2019). Polymicrobial interactions impact individual species fitness, biofilm forming capabilities, virulence factors and antimicrobial responses, all potentially compromising therapeutic success. Therefore, it is important to evaluate microbial interactions during multispecies infection.

In this study, we evaluated the direct and indirect interactions of PA and SA on Mab growth rate, biofilm formation, macrophage internalization and GPL expression to better understand the impact of microbial interactions on Mab pathogenesis and virulence (PA collectively used for *P. aeruginosa* isolates used in this study, unless specified as PaATCC for ATCC 27853). This is the first reported study to evaluate the *in vitro* interaction between Mab and SA. We observed that PaATCC and SA either co-cultured with Mab directly or indirectly reduced growth of Mab (Figures 1A,B, 2A,B). Although, the growth inhibition of Mab was maximum in presence of PaATCC. PaATCC also reduced the growth of SA, especially MSSA (Figures 1D,E). PA is known to produce virulence factors and secondary metabolites that inhibit growth of other bacterial species (Briaud et al., 2019; Jurado-Martín et al., 2021; Qin et al., 2022). We found that the inhibitory effects of PaATCC supernatant were not limited to only Mab, but extended to the other subspecies, *M. abscessus*, subsp. *massilense* and *bolettii*. The heat-inactivated supernatant of PaATCC had less of an inhibitory effect compared to the non-heat inactivated supernatant (Figures 3A–C). PaATCC supernatant prepared in LB media inhibited Mab growth (Supplementary Figure S4A); however, supernatant prepared by culturing PaATCC in 7H9 Middlebrook (MB) media did not inhibit Mab growth (Supplementary Figure S4B), similar to recent work (Idosa et al., 2022). Our experiments clearly demonstrate soluble inhibitory factors produced by PA, some of which are heat labile. These variations could be attributed to the fact that different growth conditions induce production of varied extracellular factors by PA that facilitate it to compete with other microorganisms and survive unfavorable environment (Machan et al., 1991; Hotterbeekx et al., 2017).

Polymicrobial biofilms characterize chronic lung infections, impact the disease outcome and treatment strategies (Hauser et al., 2011; Filkins and O’Toole, 2015), and significantly contribute to the virulence and antimicrobial responses of many pathogenic microorganisms (Di Domenico et al., 2017; Ciofu et al., 2022; Usui et al., 2023), including in patients with CF (Archer et al., 2011; Bjarnsholt et al., 2012; Jo, 2013; Qvist et al., 2015). Microorganisms in biofilm are in an altered metabolic state that makes them more resistant to antibiotics compared to their planktonic forms (Crabbé et al., 2019; Kumar et al., 2019; Ciofu et al., 2022; Usui et al., 2023). This is because of the biofilm’s extracellular matrix, which reduces antibiotic concentrations around the organisms (Kumar et al., 2017, 2019; Uruén et al., 2021). In our experiments, direct contact between Mab and either PA or SA inhibited biofilm forming capabilities of Mab (Figure 4A). In contrast, Mab did not inhibit biofilm formation by PA or SA (Figure 4B). This implies that the fast-growing organisms such as the PA and SA dominate during coexistence in a biofilm affecting biofilms of the slow growers as described earlier during the binary culture of fast and slow growing organisms (Banks and Bryers, 1991; Yang et al., 2008; Rodríguez-Sevilla et al., 2018a). This finding may further explain why Mab does not typically emerge in patients with CF until PA and SA are suppressed or eradicated.

While earlier studies demonstrated that the direct interaction of PA with Mab in co-culture inhibits Mab’s biofilm formation (Rodríguez-Sevilla et al., 2018a, 2019; Idosa et al., 2022), we report for the first time that even the indirect interaction (using supernatant) of PA and SA can inhibit Mab biofilm formation (Figures 5A,B). This perhaps can be implicated to the expression and production of different extracellular factors such as LasB elastase, rhamnolipids, exotoxins, phenazines and 2-Heptyl-4-hydroxyquinoline-N-oxide (HQNO) that have antimicrobial and antibiofilm properties against other microbes during coinfection (Davies et al., 2007; Sotirova et al., 2008; Hotterbeekx et al., 2017). One such secreted factor is pyocyanin, a phenazine with antimicrobial properties, and also known to inhibit biofilm formation by SA during coinfection (Hauser et al., 2011; Korgaonkar et al., 2013). We observed increased production of pyocyanin by PaATCC when co-cultured with Mab (Supplementary Figure S3B). However, in our study we found that the growth of Mab was unaffected in media supplemented with pyocyanin compared to the normal media control, though it significantly inhibited the biofilm forming capabilities of Mab (Figures 5C,D). This indicates that not only pyocyanin, but other PA exoproducts have inhibitory effect on Mab growth.

Microbial interactions during coinfection may increase virulence (Hotterbeekx et al., 2017) as demonstrated during coinfection of *P. aeruginosa* and MRSA in a porcine wound infection model, where *P. aeruginosa* enhanced MRSA internalization in lung epithelial cells even while inhibiting MRSA growth during coinfection (Pastar et al., 2013; Briaud et al., 2019). This enhanced internalization was mediated by the variety of extracellular factors produced by *P. aeruginosa* that promote bacterial invasion in host tissues causing tissue damage (Pastar et al., 2013; Hotterbeekx et al., 2017). We found that direct or indirect contact with PaATCC also increased Mab internalization by RAW264.7 macrophages (Figures 6A,C) even when PaATCC presence inhibited Mab growth during co-culture. Furthermore, Mab GPL expression was also reduced during indirect contact with PaATCC (Figure 6D). Lowered GPL levels are associated with a rough Mab morphotype, which has been associated with more severe disease (Howard et al., 2006; Catherinot et al., 2007, 2009). However, SA did not affect the invasion and GPL expression in Mab (Figures 6A,C,D). Collectively, PaATCC has complex effects upon Mab, reducing growth and biofilm formation, but also pushing Mab to adopt its rough morphotype by affecting lipid expression thereby, impacting Mab virulence.

We conducted further experiments to understand whether such effects were PA strain specific. All PA isolates during direct contact with Mab affected growth and biofilm formation by Mab (Figures 7A,C). However, not all PA isolates impacted the growth pattern and GPL expression of Mab with equal magnitude during indirect contact (Figures 7B,E), while still affecting biofilm formation (Figure 7D). Since Mab growth was not significantly affected during indirect contact with PA strains other than MPAO1 which inhibited Mab growth compared to media control but not as strongly as PaATCC, we did not check the effect of heat-inactivated supernatant of PA strains on Mab. PA isolates secrete varied exoproducts that demonstrate different properties and functions. Hence, we concluded that there is strain-specific expression of different exoproducts, supported by others’ work (Davies et al., 2007; Sotirova et al., 2008; Hotterbeekx et al., 2017).

As far as assessing the durability of PaATCC effects on Mab, we pre-exposed Mab to different concentrations of PaATCC supernatant and then again cultured Mab in standard MH media. We observed no difference in Mab growth pattern, biofilm formation and GPL expression after the supernatant was removed compared to the unexposed control (Figures 8A–C). Pre-exposure of Mab to supernatant of other PA isolates and then culture in normal MH media also showed no difference in the phenotypic characteristics of Mab compared to the control (Supplementary Figures S3A–C). This indicates that direct proximity or the indirect interaction mediated by the secretory molecules of PA alter Mab phenotypic characteristics and virulence.

## 5 Conclusion

Interaction between PA, SA and Mab is complex, and all our observations indicate that PA and SA antagonize Mab growth and biofilm formation during direct contact or indirect interaction. PA dominates, suppressing growth of the other two, but SA can also reduce Mab growth. PA favors Mab transformation to rough morphotype, and increases Mab internalization by macrophages impacting Mab virulence and facilitating severe disease conditions. Co-culture studies of these CF pathogens can therefore provide important insights into the antagonistic or synergistic behavior that can ultimately influence the virulence, pathogenesis, antibiotic responses, severity of disease outcomes. Better understanding of the complex and temporally dynamic milieu of the CF lung may lead to improved treatment strategies by anticipating effects of eradicating one species on behavior of other species.

## Data Availability

The original contributions presented in the study are included in the article/Supplementary material, further inquiries can be directed to the corresponding author.
