# Supplementary material for: Polymicrobial interactions influence Mycobacterium abscessus co-existence and biofilm forming capabilities
Source: Front Microbiol. 2024 Nov 25;15:1484510. doi: 10.3389/fmicb.2024.1484510 (PMC11627178; doi:10.3389/fmicb.2024.1484510)
Supplement: Supplementary file 1 [file Image_1.pdf]

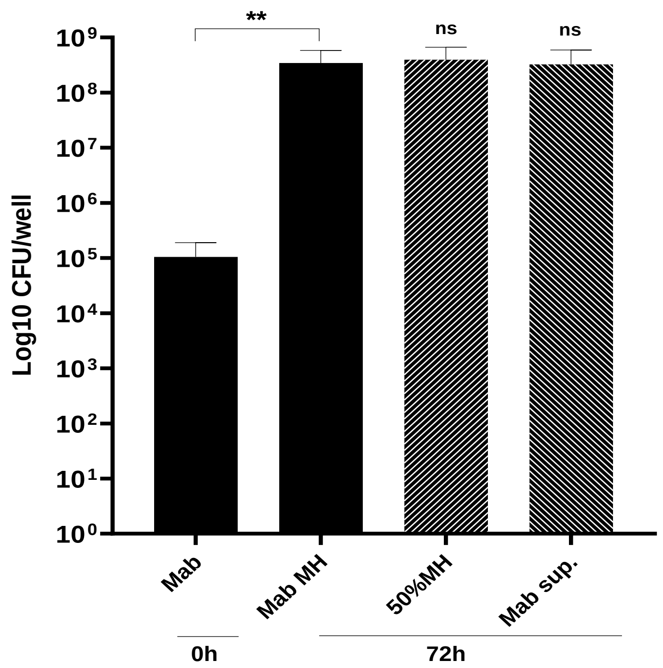

Supplementary figure 1: Comparison of Mab biofilm forming capabilities in different conditions. Mab in MH media, MH media diluted 1:1 with 1xPBS and Mab supernatant diluted 1:1 with MH media showed comparable biofilm formation after 72 h. Diluted MH media and supernatant prepared by growing Mab in MH media were used as controls to account for the use of spent media (supernatant) to study indirect interaction of Mab with *P. aeruginosa* isolates, MRSA and MSSA during indirect contact experiments. Sup. - supernatant

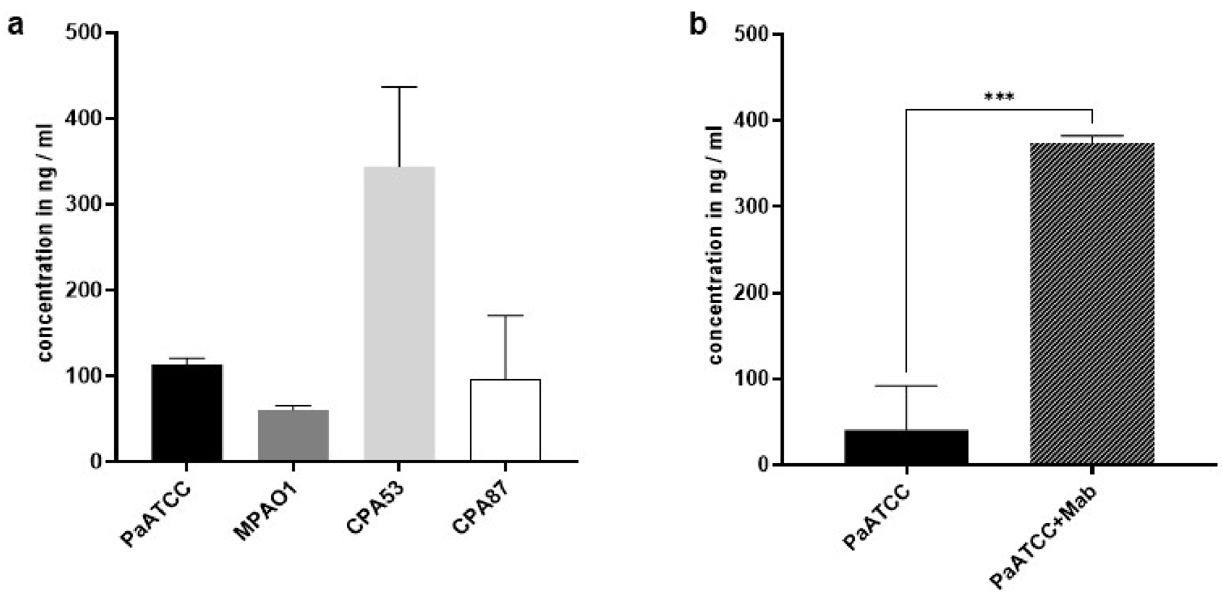

Supplementary figure 2: Pyocyanin production by different *P. aeruginosa* strains. a) Pyocyanin was extracted from the supernatants of strains of *P. aeruginosa* cultures after 24 h of incubation at 37°C in a shaking incubator. **b)** Pyocyanin production after 72 h by PaATCC in presence of Mab during direct co-culture. Significant production of pyocyanin by PaATCC was observed during co-culture with Mab compared to PaATCC monoculture. Pyocyanin concentration was measured (Abs 520nm). The data represents the mean and standard deviation of two independent experiments. Statistics were performed using Student's t-test *** = p<0.0005

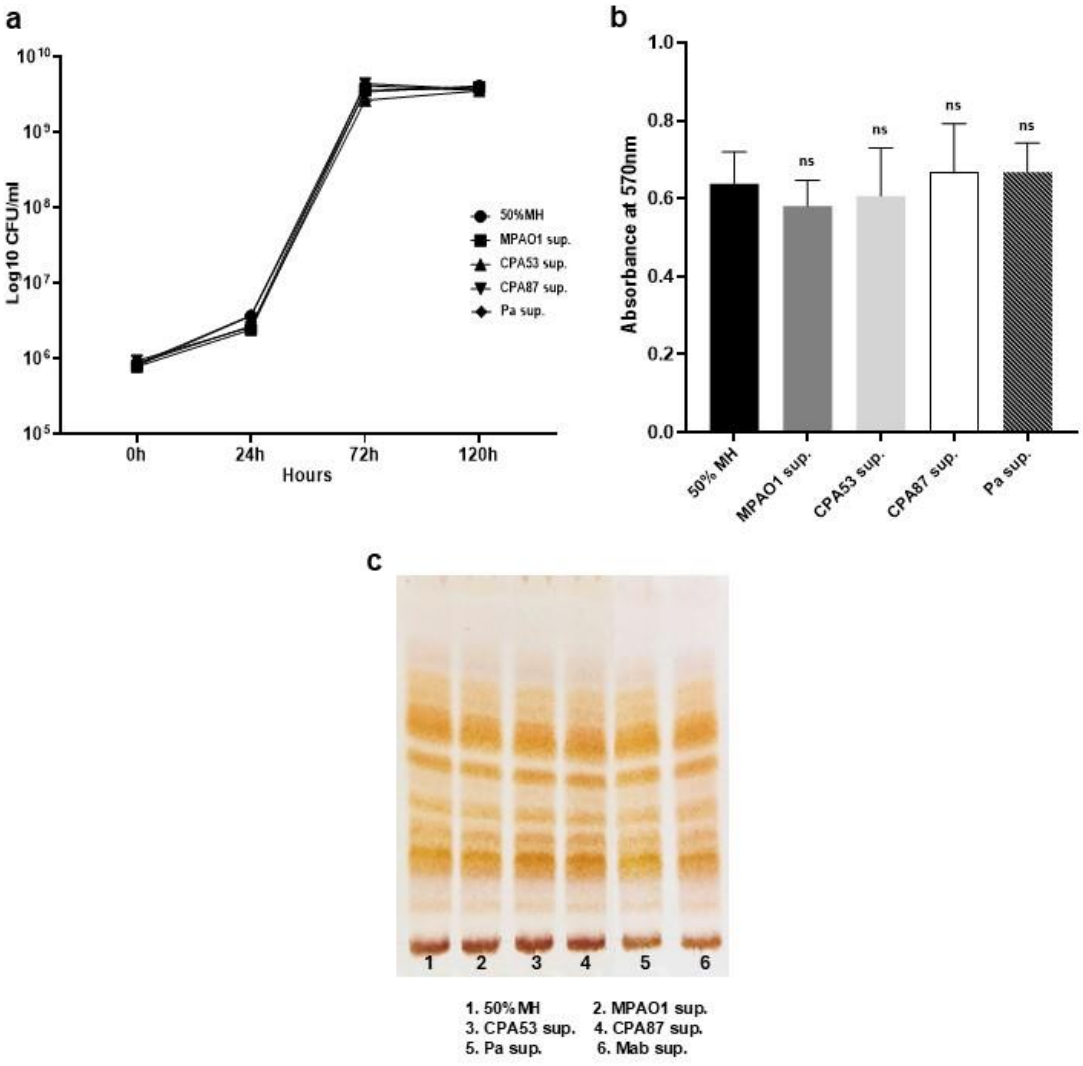

Supplementary figure 3: Mab revives its normal growth pattern, biofilm forming capabilities and GPL expression. Mab was grown in supernatant of different *P. aeruginosa* isolates (diluted 1:1 with MH media) and then centrifuged to pellet the bacteria. This pellet was resuspended in 1xPBS and used as an inoculum for growth curve, biofilm assay and for cultures to isolate GPL. Mab grown in diluted MH media (1:1 with 1xPBS) was used as a control. **a)** Mab pretreated with supernatants of different *P. aeruginosa* isolates when grown in fresh media without any supernatant revived its growth pattern comparable to the controlled growth condition (growth in diluted MH media). **b)** No significant difference was observed in the biofilm forming capabilities of Mab pretreated with supernatants of different *P. aeruginosa* compared to the control (Mab grown in diluted MH media) after 120 h of incubation. **c)** GPL extraction and thin layer

chromatography of Mab grown in normal media after pretreatment with supernatants of different *P. aeruginosa* isolates did not show any significant difference in the expression of GPL as compared to the control (Mab grown in diluted MH media). The growth curve data represents the mean and standard deviation of two independent experiments plated each time in duplicate. Biofilm forming assay was performed three times, each with at least six replicates each time. The TLC is the representative image of three different experiments. Statistics were performed using one-way ANOVA for multiple comparisons. ns – represents non-significance. Sup. - supernatant

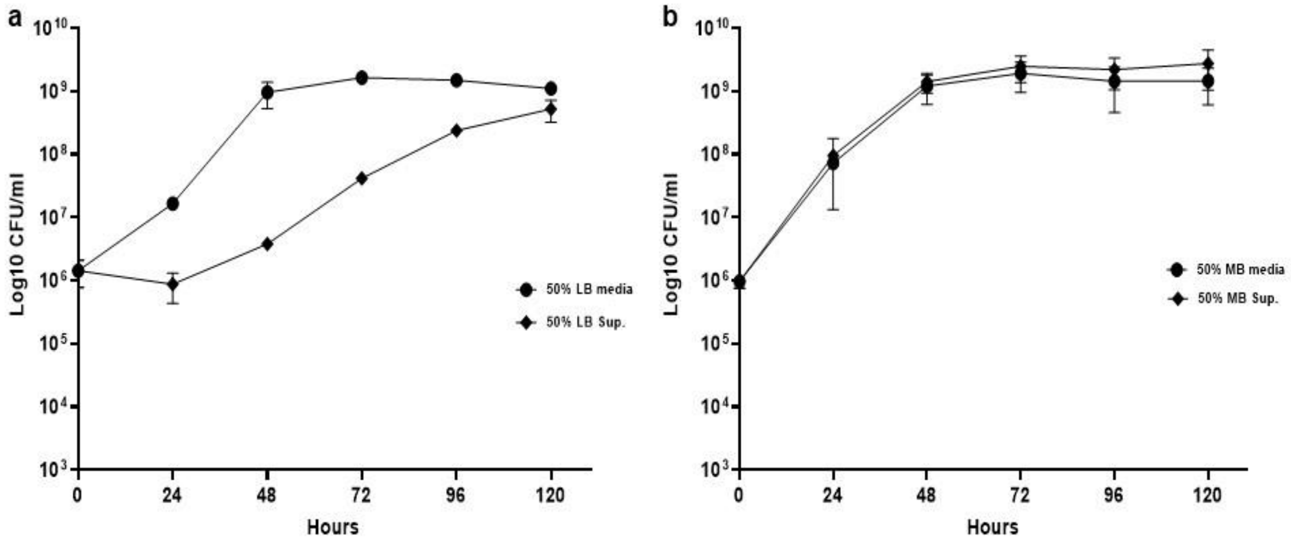

Supplementary figure 4: Growth pattern of Mab in PaATCC supernatant (indirect contact). **a)** The supernatant of PaATCC when prepared in LB media (diluted 1:1 with MH media for growth curve experiment) also demonstrated inhibitory effect on Mab growth compared to the control (Mab grown in 1:1 diluted LB media with 1xPBS). However, after the prolonged incubation (120 h) Mab managed to grow in PaATCC LB supernatant. **b)** PaATCC supernatant prepared by culturing PaATCC in MB media did not show any inhibitory effect on Mab growth compared to the control (MB media diluted 1:1 with 1XPBS). The growth curve data represents the mean and standard deviation of two independent experiments plated each time in duplicate. Sup. - supernatant
