# Supplementary material for: Polymicrobial interactions influence Mycobacterium abscessus co-existence and biofilm forming capabilities
Source: Front Microbiol. 2024 Nov 25;15:1484510. doi: 10.3389/fmicb.2024.1484510 (PMC11627178; doi:10.3389/fmicb.2024.1484510)
Supplement: Supplementary file 2 [file Table_1.pdf]

Supplementary table S1: List of organisms used in this study.

S. No	Organism	Abbreviation used	Sub species	Source
1.	*Mycobacterium abscessus* ATCC19977	Mab	*M. abscessus subspecies abscessus*	American type culture collection (ATCC)
2.	*Mycobacterium massiliense*	Mms	*M. abscessus subspecies massiliense*	Children's Hospital Los Angeles, Los Angeles, California USA
3.	NR-4266 *Mycobacterium abscessus* MC1518	Mbl1518	*M. abscessus subspecies bolletii.*	Biodefense and Emerging Infections Research Resources Repository (BEI Resources)
4.	*Pseudomonas aeruginosa* ATCC 27853	PaATCC	-	American type culture collection (ATCC)
5.	Methicillin-resistant *Staphylococcus aureus* ATCC 43300	MRSA	-	American type culture collection (ATCC)
6.	Methicillin sensitive *Staphylococcus aureus* ATCC 25923	MSSA	-	American type culture collection (ATCC)
7.	*Pseudomonas aeruginosa* MPAO1	MPAO1	Wildtype PAO1	Dr. Burgener Lab, Children's Hospital Los Angeles, Los Angeles, California USA
8.	*Pseudomonas aeruginosa* CPA53	CPA53	Clinical isolate of *Pseudomonas*	Dr. Burgener Lab, Children's Hospital Los Angeles, Los Angeles, California USA
9.	*Pseudomonas aeruginosa* CPA87	CPA87	Clinical isolate of *Pseudomonas*	Dr. Burgener Lab, Children's Hospital Los Angeles, Los Angeles, California USA
