# Supplementary material for: Polymicrobial interactions influence Mycobacterium abscessus co-existence and biofilm forming capabilities
Source: Front Microbiol. 2024 Nov 25;15:1484510. doi: 10.3389/fmicb.2024.1484510 (PMC11627178; doi:10.3389/fmicb.2024.1484510)
Supplement: Supplementary file 3 [file Table_2.pdf]

Supplementary Table 2

Supplementary Table 2A:

Comparison of Mab growth in MH media vs Mab growth in co-culture with PaATCC, MSSA and MRSA at different time points during growth curve experiment (Figure 1A and 1B).

For Figure 1A - Mab initial inoculum 10^5			
Time points (In hours)	Mab vs Mab + PaATCC	Mab vs Mab + MRSA	Mab vs Mab + MSSA
24	**	***	ns
48	**	**	**
72	*	*	*
96	***	***	***
120	**	**	**

For Figure 1B - Mab initial inoculum 10^7			
Time points (In hours)	Mab vs Mab + PaATCC	Mab vs Mab + MRSA	Mab vs Mab + MSSA
24	**	*	**
48	***	***	***
72	***	***	***
96	****	****	****
120	*	*	ns

# Statistical analysis of growth pattern of Mab ($5x10^5$ CFU/ml and $5x10^7$ CFU/ml), in co-culture (direct contact) with PaATCC, MRSA and MSSA. The starting inoculum for PaATCC, MRSA and MSSA was 10^3 for each, respectively (Mab5, represents $5x10^5$ CFU/ml and Mab7, represents $7x10^7$ CFU/ml respectively). Growth curve experiments were performed in MH media as it supported the growth of all the participating bacteria in a co-culture. The analysis was performed by comparing the growth control (Mab) versus growth in co-culture using the data of three independent experiments plated each time in duplicate. We performed statistical analyses using Prism Software (GraphPad version 9.0.0), and a p-value less than or equal to 0.05 was considered statistically significant. Independent Student's t-test was used for comparison between two conditions. **** = p<0.0001, *** = p<0.0005, and ** = p<0.005. * = p<0.05, ns – non-significance.

Supplementary Table 2B:

Comparison of PaATCC growth in MH media vs PaATCC growth in co-culture with Mab ($5x10^5$ CFU/ml and $5x10^7$ CFU/ml), MRSA and MSSA at different time points during growth curve experiment (Figure 1C).

**For Figure 1C- PaATCC initial inoculum 10^3**				
**Time points (In hours)**	**PaATCC vs PaATCC + Mab5**	**PaATCC vs PaATCC + Mab7**	**PaATCC vs PaATCC + MRSA**	**PaATCC vs PaATCC + MSSA**
**24**	ns	ns	ns	ns
**48**	ns	ns	ns	ns
**72**	ns	ns	ns	ns
**96**	ns	ns	ns	ns
**120**	ns	ns	**	ns

# Statistical analysis of growth pattern of PaATCC in co-culture (direct contact) with Mab ($5x10^5$ CFU/ml and $5x10^7$ CFU/ml), MRSA and MSSA. The starting inoculum for PaATCC, MRSA and MSSA was 10^3 for each, respectively (Mab5, represents $5x10^5$ CFU/ml and Mab7, represents $7x10^7$ CFU/ml respectively).Growth curve experiments were performed in MH media as it supported the growth of all the participating bacteria in a co-culture. The analysis was performed by comparing the growth control versus growth in co-culture using the data of three independent experiments plated each time in duplicate. We performed statistical analyses using Prism Software (GraphPad version 9.0.0), and a p-value less than or equal to 0.05 was considered statistically significant. Independent Student's t-test was used for comparison between two conditions. **** = p<0.0001, *** = p<0.0005, and ** = p<0.005. * = p<0.05, ns – non-significance.

Supplementary Table 2C:

Comparison of MSSA and MRSA growth in MH media vs growth in co-culture with Mab ($5x10^5$ CFU/ml and $5x10^7$ CFU/ml) and PaATCC at different time points during growth curve experiment (Figures 1D and 1E).

**For Figure 1D – MSSA initial inoculum 10^3**			
**Time points (In hours)**	**MSSA vs MSSA + Mab5**	**MSSA vs MSSA + Mab7**	**MSSA vs MSSA + PaATCC**
**24**	ns	ns	Ns
**48**	ns	ns	**
**72**	ns	ns	***
**96**	ns	ns	***
**120**	*	ns	**

For Figure 1E – MRSA initial inoculum 10^3			
Time points (In hours)	MRSA vs MRSA + Mab5	MRSA vs MRSA + Mab7	MRSA vs MRSA + PaATCC
24	ns	ns	Ns
48	ns	ns	Ns
72	ns	ns	Ns
96	**	**	Ns
120	**	**	Ns

# Statistical analysis of growth pattern of MSSA and MRSA in co-culture (direct contact) with Mab ($5x10^5$ CFU/ml and $5x10^7$ CFU/ml) and PaATCC. The starting inoculum for PaATCC, MRSA and MSSA was 10^3 for each, respectively (Mab5, represents $5x10^5$ CFU/ml and Mab7, represents $7x10^7$ CFU/ml respectively).Growth curve experiments were performed in MH media as it supported the growth of all the participating bacteria in a co-culture. The analysis was performed by comparing the growth control versus growth in co-culture using the data of three independent experiments plated each time in duplicate. We performed statistical analyses using Prism Software (GraphPad version 9.0.0), and a p-value less than or equal to 0.05 was considered statistically significant. Independent Student's t-test was used for comparison between two conditions. **** = p<0.0001, *** = p<0.0005, and ** = p<0.005. * = p<0.05, ns – non-significance.

Supplementary Table 3

Comparison of Mab growth in 50% MH media vs Mab growth in supernatant (indirect contact) of PaATCC, MRSA and MSSA at different time points during growth curve experiment (Figure 2A and 2B).

For Figure 2A - Mab initial inoculum 10^5			
Time points (In hours)	Mab in 50%MH vs Mab in PaATCC Sup.	Mab in 50%MH vs Mab in MRSA Sup.	Mab in 50%MH vs Mab in MSSA Sup.
24	**	Ns	**
48	***	**	***
72	***	***	***
96	***	***	***
120	****	***	***

For Figure 2B - Mab initial inoculum 10^7			
**Time points (In hours)**	**Mab in 50%MH vs Mab in PaATCC Sup.**	**Mab in 50%MH vs Mab in MRSA Sup.**	**Mab in 50%MH vs Mab in MSSA Sup.**
**24**	*	Ns	*
**48**	***	**	***
**72**	***	***	***
**96**	**	*	*
**120**	**	*	*

# Statistical analysis of growth pattern of Mab (5x10^5 CFU/ml and 5x10^7 CFU/ml) in supernatant (indirect contact) of PaATCC, MRSA and MSSA respectively. The starting inoculum of Mab was 5x10^5 CFU/ml and 7x10^7 CFU/ml respectively. Growth curve experiments were performed in 50% diluted MH media and 50% diluted supernatant. The analysis was performed by comparing the growth control versus growth in supernatant using the data of three independent experiments plated each time in duplicate. We performed statistical analyses using Prism Software (GraphPad version 9.0.0), and a p-value less than or equal to 0.05 was considered statistically significant. Independent Student's t-test was used for comparison between two conditions. **** = p<0.0001, *** = p<0.0005, and ** = p<0.005. * = p<0.05, ns – non-significance.

Supplementary Table 4

Comparison of Mab, Mms and Mbl1518 growth in 50% MH media vs growth in different conditions (MH media, PaATCC supernatant and heat inactivated PaATCC supernatant at different time points during growth curve experiment (Figure 3A, 3B and 3C).

For Figure 3A - Mab initial inoculum 10^5			
**Time points (In hours)**	**Mab in 50%MH vs Mab in MH media**	**Mab in 50%MH vs Mab in PaATCC Sup.**	**Mab in 50%MH vs Mab in Heat inactivated PaATCC Sup.**
**24**	Ns	***	**
**48**	Ns	***	**
**72**	Ns	****	**
**96**	Ns	****	Ns
**120**	ns	****	Ns

For Figure 3B - Mms initial inoculum 10^5			
**Time points (In hours)**	**Mab in 50%MH vs Mab in MH media**	**Mab in 50%MH vs Mab in PaATCC Sup.**	**Mab in 50%MH vs Mab in Heat inactivated PaATCC Sup.**
**24**	ns	***	Ns
**48**	ns	****	**
**72**	ns	****	**
**96**	ns	****	**
**120**	ns	****	Ns

For Figure 3C – Mbl1518 initial inoculum 10^5			
**Time points (In hours)**	**Mab in 50%MH vs Mab in MH media**	**Mab in 50%MH vs Mab in PaATCC Sup.**	**Mab in 50%MH vs Mab in Heat inactivated PaATCC Sup.**
**24**	ns	ns	ns
**48**	ns	****	**
**72**	ns	****	**
**96**	ns	***	**
**120**	ns	***	*

# Statistical analysis of growth pattern of Mab, Mms and Mbl1518 in non-heat inactivated and heat inactivated PaATCC supernatant (indirect contact) respectively. The starting inoculum of Mab, Mms and Mbl1518 was $5x10^5$ CFU/ml and $7x10^7$ CFU/ml respectively. Growth curve experiments were performed in 50% diluted non-heat inactivated and heat inactivated PaATCC supernatant respectively. The analysis was performed by comparing the growth control versus growth in different conditions mentioned above using the data of two independent experiments plated each time in duplicate. We performed statistical analyses using Prism Software (GraphPad version 9.0.0), and a p-value less than or equal to 0.05 was considered statistically significant. Independent Student's t-test was used for comparison between two conditions. **** = p<0.0001, *** = p<0.0005, and ** = p<0.005. * = p<0.05, ns – non-significance.

Supplementary Table 5

Comparison of Mab growth in 50% MH media vs growth in 50% MH media supplemented with different concentrations of commercially available pyocyanin during growth curve experiment (Figure 5C).

For Figure 5C- Mab initial inoculum 10^5				
**Time points (In hours)**	**Mab 50% MH vs Mab 50% MH+200µg/ml Pyocyanin**	**Mab 50% MH vs Mab 50% MH+150µg/ml Pyocyanin**	**Mab 50% MH vs Mab 50% MH+100µg/ml Pyocyanin**	**Mab 50% MH vs Mab 50% MH+DMSO (Vol. equivalent to pyocyanin added for 200µg/ml concentration)**
**24**	ns	ns	Ns	ns
**48**	*	ns	Ns	ns
**72**	ns	ns	Ns	ns
**96**	ns	ns	Ns	ns
**120**	ns	ns	Ns	ns

# Statistical analysis of growth pattern of Mab in presence of different concentration of pyocyanin during growth curve. The starting inoculum of Mab was $5x10^5$ CFU/ml. Growth curve experiments were performed in 50% diluted MH media supplemented with different concentration of pyocyanin (200, 150 and 100 µg/ml respectively). The analysis was performed by comparing the growth control versus growth in different conditions mentioned above using the data of two independent experiments plated each time in duplicate. We performed statistical analyses using Prism Software (GraphPad version 9.0.0), and a p-value less than or equal to 0.05 was considered statistically significant. Independent Student's t-test was used for comparison between two conditions. **** = p<0.0001, *** = p<0.0005, and ** = p<0.005. * = p<0.05, ns – non-significance.

Supplementary Table 6

Comparison of Mab growth in MH media vs Mab growth in co-culture with different *Pseudomonas aeruginosa* strains at different time points during growth curve experiment (Figure 7A).

For Figure 7A – Mab initial inoculum 10^5			
Time points (In hours)	Mab vs Mab + MPAO1	Mab vs Mab + CPA53	Mab vs Mab + CPA87
24	***	***	***
48	****	****	****
72	****	****	****
96	****	****	****
120	****	****	****

# Statistical analysis of growth pattern of Mab ($5x10^5$ CFU/ml) in co-culture (direct contact) with different *Pseudomonas aeruginosa* strains. The starting inoculum of *Pseudomonas aeruginosa strains* was 10^3 for each, respectively. Growth curve experiments were performed in MH media as it supported the growth of all the participating bacteria in a co-culture. The analysis was performed by comparing the growth control (Mab) versus growth in co-culture using the data of two independent experiments plated each time in duplicate. We performed statistical analyses using Prism Software (GraphPad version 9.0.0), and a p-value less than or equal to 0.05 was considered statistically significant. Independent Student's t-test was used for comparison between two conditions. **** = p<0.0001, *** = p<0.0005, and ** = p<0.005. * = p<0.05, ns – non-significance.

Supplementary Table 7

Comparison of Mab growth in 50% MH media vs Mab growth in supernatant (indirect contact) of other *Pseudomonas aeruginosa* strains (MPAO1, CPA53 and CPA87) at different time points during growth curve experiment (Figure 7B).

For Figure 7B – Mab initial inoculum 10^5				
Time points (In hours)	Mab in 50%MH vs Mab in 50% MPAO1 Sup.	Mab in 50%MH vs Mab in 50% CPA53 Sup.	Mab in 50%MH vs Mabin 50% CPA87 Sup.	Mab in 50%MH vs Mabin 50% PaATCC Sup.
24	ns	ns	ns	*
48	***	**	ns	***
72	***	**	**	****
96	**	ns	ns	***
120	**	ns	ns	***

# Statistical analysis of growth pattern of Mab (5x10^5 CFU/ml) in supernatant (indirect contact) of *Pseudomonas aeruginosa* isolates MPAO1, CPA53 and CPA87 respectively. Growth curve experiments were performed in 50% diluted MH media and 50% diluted supernatant. The analysis was performed by comparing the growth control versus growth in supernatant using the data of two independent experiments plated each time in duplicate. We performed statistical analyses using Prism Software (GraphPad version 9.0.0), and a p-value less than or equal to 0.05 was considered statistically significant. Independent Student's t-test was used for comparison between two conditions. **** = $p<0.0001$, *** = $p<0.0005$, and ** = $p<0.005$. * = $p<0.05$, ns – non-significance.

Supplementary Table 8

Comparison of Mab growth in 50% MH media after pretreatment with different concentrations (50%, 25% and 10%) of PaATCC supernatant at different time points during growth curve experiment (Figure 8A).

For Figure 8A – Mab initial inoculum 10^5			
Time points (In hours)	Mab pretreated with 50%MH vs Mab pretreated with 50% PaATCC Sup.	Mab pretreated with 50%MH vs Mab pretreated with 25% PaATCC Sup	Mab pretreated with 50%MH vs Mab pretreated with 10% PaATCC Sup
24	***	**	ns
48	ns	ns	ns
72	ns	ns	ns
96	ns	ns	ns
120	ns	ns	ns

# Statistical analysis of growth pattern of Mab (5x10^5 CFU/ml) in 50% MH media after pretreatment with different concentrations of PaATCC supernatant (50%, 25% and 10% respectively). Mab was grown in different concentrations of PaATCC supernatant and then centrifuged. The Mab pellet after centrifugation resuspended in 1XPBS was used as an initial inoculum for growth curve experiment. Growth curve experiments were performed in 50% diluted MH media. The analysis was performed by comparing the growth in MH media versus growth of pretreated Mab in MH media using the data of three independent experiments plated each time in duplicate. We performed statistical analyses using Prism Software (GraphPad version 9.0.0), and a p-value less than or equal to 0.05 was considered statistically significant. Independent Student's t-test was used for comparison between two conditions. **** = $p<0.0001$, *** = $p<0.0005$, and ** = $p<0.005$. * = $p<0.05$, ns – non-significance.
